# Interhemispheric connectivity measured with transcranial magnetic stimulation and EEG as an objective marker of cognitive risk

**DOI:** 10.1093/braincomms/fcag251

**Published:** 2026-07-15

**Authors:** Noa Zifman, Hilla Fogel, Riki Rosenberg, Ofri Levy-Lamdan, Nastya Verhovski, Tzipora Strauss, Evelyne Bischof, Abigail Goshen

**Affiliations:** Clinical Research Department, QuantalX Neuroscience Ltd, Kfar-Saba 4453001, Israel; Clinical Research Department, QuantalX Neuroscience Ltd, Kfar-Saba 4453001, Israel; Sheba Longevity Center, Sheba Medical Center, Ramat-Gan 52621, Israel; Clinical Research Department, QuantalX Neuroscience Ltd, Kfar-Saba 4453001, Israel; Clinical Research Department, QuantalX Neuroscience Ltd, Kfar-Saba 4453001, Israel; Sheba Longevity Center, Sheba Medical Center, Ramat-Gan 52621, Israel; Gray Faculty of Medicine, Tel Aviv University, Tel-Aviv 6997801, Israel; Sheba Longevity Center, Sheba Medical Center, Ramat-Gan 52621, Israel; Gray Faculty of Medicine, Tel Aviv University, Tel-Aviv 6997801, Israel; Sheba Longevity Center, Sheba Medical Center, Ramat-Gan 52621, Israel; Department of Management, Health Management Program, Bar-Ilan University, Ramat Gan 5290002, Israel

**Keywords:** interhemispheric connectivity, cognitive impairment, transcranial magnetic stimulation evoked potentials

## Abstract

Cognitive impairment is a common complaint in older adults, yet its evaluation relies largely on subjective reports and cognitive tests with variable sensitivity. Identifying objective neurophysiological markers of brain function may enable earlier detection and more individualized intervention strategies. A total of 454 participants (mean age 61.4 ± 7.5 years; 45.3% females) underwent bilateral dorsolateral prefrontal cortex (DLPFC) transcranial magnetic stimulation (TMS)-EEG with the Delphi-MD system, alongside cognitive testing (Monreal Cognitive Assessment [MoCA] and NeuroTrax domains: memory, attention and executive function) and emotional inventories. Linear regression tested demographic and clinical predictors of cognition. K-means clustering identified cognitive subgroups, with between-cluster interhemispheric connectivity (IHC) differences assessed by Mann–Whitney U-tests. Binary logistic regression with 5000 bootstrap resamples evaluated IHC as a predictor of cognitive cluster membership. Older age was associated with lower MoCA (worse) (*P* < 0.001), while male sex associated with worse verbal memory (*P* < 0.001); no factors were associated with attention or executive function. K-means clustering yielded two groups based on MoCA and domain scores: cognitively normal (CN, *n* = 367) and cognitively impaired (CI, *n* = 68), differing across all domains (all *P* < 0.001). CI participants displayed reduced DLPFC IHC (*P* < 0.001). Logistic regression confirmed that reduced IHC strongly predicted impairment: one-unit decrease in right DLPFC IHC corresponded to approximately 4.35-fold higher odds of impairment (reciprocal OR = 4.35, 95% CIboot = 1.82–10.00), while a one-unit decrease in left DLPFC IHC corresponded to 3.03-fold higher odds of impairment (reciprocal OR = 3.03, 95% CIboot = 1.30–6.67). These effects were independent of age and sex. Reduced DLPFC IHC measured by TMS-EEG is robustly associated with increased risk of cognitive impairment, supporting its potential as an objective, early neurophysiological biomarker quantifying cognitive decline.

## Teaser

Zifman *et al*. demonstrate that prefrontal interhemispheric connectivity measured with transcranial magnetic stimulation and EEG provides an objective marker of cognitive performance in community-dwelling adults. Reduced interhemispheric connectivity robustly distinguished cognitively impaired individuals independently of age and sex, highlighting its clinical utility for detection and risk stratification in ageing populations.

## Graphical Abstract

**Graphical Abstract:**
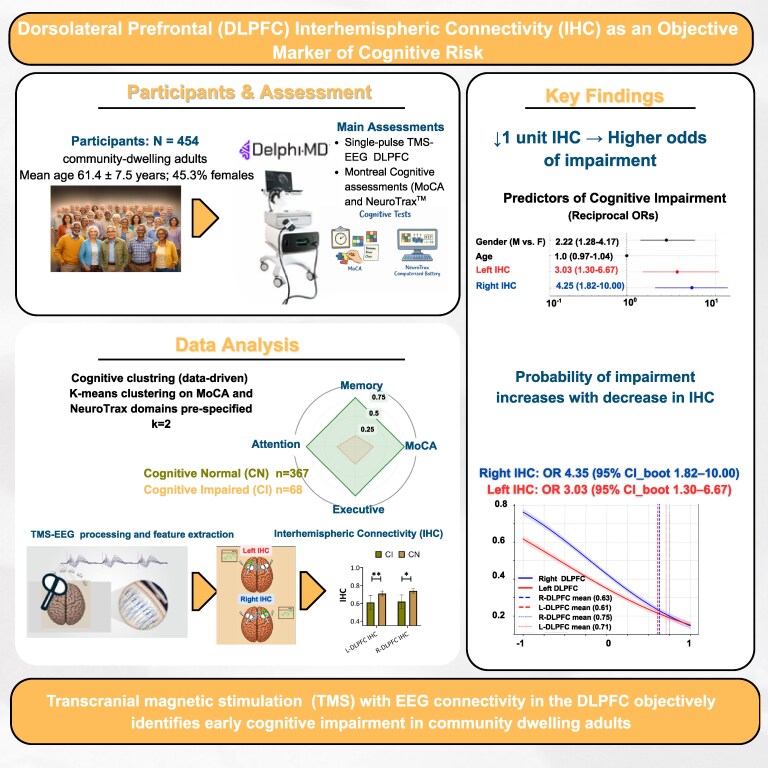

## Introduction

Cognitive impairment and dementia represent major public health challenges in ageing populations, with prevalence increasing markedly after midlife and imposing substantial personal and societal costs.^1,2^ Early identification of individuals at risk remains a priority, as conventional screening tools such as the Montreal Cognitive Assessment (MoCA) provide global measures of cognition but have limited sensitivity to early or subtle network dysfunction.^3^ Neurobiological models of ageing emphasize that cognitive decline reflects not only localized pathology but also large-scale network inefficiency, particularly in prefrontal circuits that mediate executive control, working memory, and goal-directed behaviour.^4,5^

The dorsolateral prefrontal cortex (DLPFC) is a strategic hub, subserving higher-order cognition and exerting top-down modulation over distributed cortical and subcortical targets.^6^ Age-related alterations in interhemispheric coordination of the DLPFC are well documented, including reductions in effective connectivity and compensatory hemispheric recruitment described in frameworks such as HAROLD (Hemispheric Asymmetry Reduction in Older Adults).^7^ Low-frequency repetitive transcranial magnetic stimulation (rTMS) over right DLPFC (R-DLPFC) and high-frequency rTMS over left DLPFC (L-DLPFC) have been reported to significantly improve memory function.^8^ Disrupted prefrontal communication has been observed in mild cognitive impairment (MCI) and Alzheimer’s disease, where abnormal patterns of inter-hemispheric and intra-hemispheric activation correlate with poorer cognitive outcomes.^9,10^ These findings suggest that measures of prefrontal connectivity may index physiologic cognitive reserve and reveal early markers of decline.

TMS combined with electroencephalography (TMS-EEG) offers a causal, time-resolved probe of cortical effective connectivity by perturbing one region and measuring evoked responses across the network.^11–14^ Unlike correlational neuroimaging approaches, TMS-EEG can directly quantify the efficiency of circuit communication, making it a promising tool for identifying biomarkers of cognitive health. Prior work has demonstrated that prefrontal TMS-EEG indices are sensitive to ageing, neuropsychiatric disorders and neuromodulatory interventions.^15–17^ However, the potential of DLPFC interhemispheric connectivity (IHC) as a biomarker for cognitive impairment and cognitive reserve in non-clinical, community-dwelling populations has not been systematically evaluated.

The present study addresses this gap by deriving an IHC index from DLPFC TMS-EEG in a large community-based sample and testing its association with data-driven cognitive clusters. We hypothesized that reduced prefrontal IHC would characterize individuals with impaired cognition independent of age, sex, or mood symptoms, and that this index would outperform conventional demographic and clinical predictors. We aim to establish DLPFC IHC as a robust, non-invasive, and physiologically grounded indicator of cognitive impairment and cognitive reserve in aging populations.

## Materials and methods

### Study design and population

In this cross-sectional prospective study, neurologically and psychiatrically healthy subjects at the ages of 40–90 were consecutively recruited at the Sheba Longevity Center. Study participants were enrolled based on the following eligibility criteria. Inclusion criteria: Male and female Subjects at the ages of 40–90. Exclusion criteria: (1) Previous diagnosis of neurodegenerative diseases such as Alzheimer's disease, Parkinson's disease, Lewy body dementia; (2) Previous diagnosis of psychiatric disease; (3) Currently undergoing chemotherapy or radiation therapy. (4) Participants with a history of organic brain lesions (e.g. stroke).

### Cognitive and clinical assessments

Subjects underwent global cognition assessment with the MoCA, a widely used screening tool sensitive to MCI and dementia, validated across diverse populations^3^ and adjusted to education. Domain-specific cognitive functions were measured using the NeuroTrax computerized battery (Mindstreams), which provides age and education adjusted scores for verbal memory, attention and executive function, and has been validated in ageing and clinical cohorts.^18,19^ Depressive and anxiety symptoms were assessed using the Beck Depression Inventory-II (BDI-II) and the Beck Anxiety Inventory (BAI), respectively, both of which are reliable self-report measures with strong psychometric validation in clinical and non-clinical populations.^20,21^ In addition, subjective reports of cognitive decline (SCD) were collected.

### TMS-EEG procedure

TMS-EEG evaluation was performed utilizing Delphi-MD (Direct Electro-Physiological Imaging medical device) system (QuantalX Neuroscience Ltd.) which is an integrated system that records, elicits and quantifies TMS-evoked potentials (TEPs). The Delphi-MD magnetic stimulation is a biphasic waveform and includes a figure-8 coil, recording amplifier is DC coupled, with sampling rate of 5 kHz, Delphi-MD evaluation was performed in rested awake subjects, with eyes closed, earplugs were used to mask noises and protect hearing. Delphi-MD electrode cap includes 10–20 system 32 scalp pallet Ag/AgCl electrodes, with ground and reference electrodes positioned on the ear lobes. Data recording and analysis was performed by the Delphi-MD software (version 1.0). The software includes automated online data checks, alert system as well as offline pre-processing, feature extraction and production of results, described elsewhere.^22–28^ The stimulation protocol included single pulse stimulation (0.1 Hz) set to 85% of the patient rest motor threshold (RMT), with 18 repetitions per stimulation site. RMT was detected by visual indication of motor response (twitch) in the abductor pollicis brevis (ABP) of the contralateral hand, 5 out of 10 stimulations^29^ and localization of the coil over DLPFC in accordance to anatomical landmarks.^30^

The TMS coil was positioned over the left and right DLPFC according to anatomical landmarks aligned with the EEG 10–20 system,^31^ corresponding to electrodes F3 and F4, respectively. The coil was oriented at a 45° angle towards the contralateral forehead throughout stimulation.

### TEP pre-processing

All pre-processing and quality control procedures were performed automatically by Delphi software (Ver 1.0). Pre-processing includes filtering to an effective 1–45 Hz passband,^32^ powerline noise removal and TMS decay artefact removal using independent component analysis decomposition.^33–35^ Electrooculography artefacts are corrected using canonical correlation analysis (CCA) which uses maximization of linear independence.^36–38^ Data segments with high noise levels are automatically detected through processing stages using thresholding in the temporal and spectral domains. Trials with excessive noise are discarded, and noisy channels are interpolated using data from neighbouring channels. Datasets were excluded when trial rejection exceeded 30% or when insufficient spatial coverage remained. Real-time alerts were provided to the operator for datasets classified as insufficient or noisy.

### Interhemispheric connectivity index

**Figure 1:**
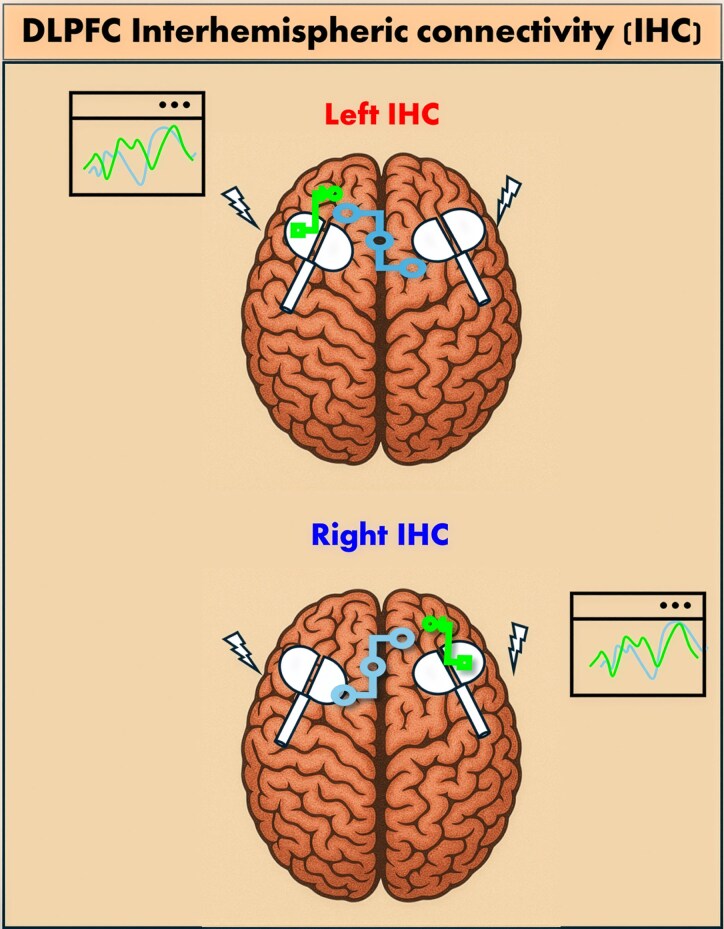
**Illustration of the measurement of L-DLPFC and R-DLPFC IHC.** Comparison of the waveform is measured from right electrodes for left dorsolateral prefrontal cortex (L-DLPFC) IHC (upper panel), and from left electrodes, for right dorsolateral prefrontal cortex R-DLPFC IHC (lower panel), in response to both L-DLPFC and R-DLPFC stimulations.

IHC was quantified as the similarity between TEPs elicited by left and right DLPFC stimulation (Fig. 1). For each electrode *c*, the trial-averaged TEP waveforms within the time window *t*  ∈ [45–200] ms post-stimulus were extracted for left $\left(x_{c}^{L}(t)\right)$ and right $(x_{c}^{R}(t))\;$ stimulation.

Channel-wise connectivity was defined as the Pearson correlation coefficient between these waveforms:


$$r_{c}=\mathrm{corr}(x_{c}^{L}(t),x_{c}^{R}(t))$$

where the correlation is computed across all sampled time points within the defined window.

IHC was then computed by averaging $r_{c}$ across predefined electrode sets for each hemisphere. The left IHC was calculated over $C_{L}=\{CZ,C3,C5,FC1\}$, and the right IHC over $C_{R}=\{CZ,C4,C6,FC2\}$:


$$\mathrm{IH}C_{\mathrm{left}}=\frac{1}{|C_{L}|}\underset{c\in C_{L}}{\sum }\;r_{c}\;\;\;\;\mathrm{IH}C_{\mathrm{right}}=\frac{1}{|C_{R}|}\underset{c\in C_{R}}{\sum }\;r_{c}$$

where the summation is performed over all electrodes in each respective set.

Central electrode regions of interest were selected due to their relative resistance to physiological artefacts (e.g. ocular activity, alpha oscillations) and their more stable evoked responses.

### Statistical analysis

Independent *t*-tests or one-way ANOVA were used to compare continuous measures such as age. Comparison of proportions was done with Chi-square or Fisher's exact test as appropriate. Where the dependent variable displayed non normal distribution non-parametric tests were applied (Mann–Whitney U-test, Kruskal-–Wallis).

### Predictability of cognitive performance by demographics and clinical features

The probability of demographic and clinical features (such as age, sex, subjective cognitive complaint, depression and anxiety) to predict cognitive performance as measured by total MoCA score, computerized memory, attention or executive function was assessed using multivariate linear regression.

### Subgrouping by cognitive performance

Given the generally healthy nature of the cohort and the use of multiple cognitive assessment tools, cognitive performance was characterized by using an unsupervised clustering approach. This data-driven method was applied to identify distinct cognitive performance profiles across domains, without relying on predefined diagnostic cut-offs. Cognitive tests’ hard thresholds, intended for screening, are often insensitive to milder cognitive deficits, with conflicting results and variability between different types of cognitive tests. To incorporate measured cognitive scores into a single comprehensive cognitive profile we used *k*-means clustering (SPSS Quick Cluster) for creating two cognitive subgroups (cognitively normal [CN] and cognitively impaired [CI]) considering scores of MoCA, memory, attention and executive function scores (listwise deletion for missingness). We pre-specified *k* = 2 with squared Euclidean distance, default initialization and convergence at the default iteration criterion, saving cluster membership and distance-to-centre for each participant. Final centroids and cluster sizes are reported.

### Interhemispheric connectivity prediction of cognitive status

Prediction of cognitive clusters by DLPFC IHC was evaluated using a binary logistic regression ORs with 95% confidence intervals were estimated from the regression coefficients, and bootstrapping with 5000 samples was used to obtain robust confidence intervals. ORs <1 were inverted (rOR = 1/OR) so all effects are ≥1, simplifying interpretation while preserving magnitude. Confidence intervals were inverted accordingly. Predicted probabilities of cognitive impairment were calculated from the fitted regression models and plotted against observed IHC values for the L-DLPFC and R-DLPFC separately. Curves were generated to visualize the risk of impairment across the full IHC range, with shaded bands indicating the 95% confidence interval. Predicted probability curves were generated from logistic regression equations using point estimates and 95% bootstrap confidence intervals (5000 samples). These curves illustrate the risk of cognitive impairment across the entire range of IHC values, with shaded bands representing the uncertainty in the estimates. We treated the left and right IHC as separate models since we were interested to reveal whether a specific direction of IHC is associated with cognitive impairment, which would be obscured by a combined metric.

Analysis was performed with SPSS version 31.0 and python3 and plots were created with the graph prism version 10.6 and Python3.

## Results

454 participants (mean age 61.4 ± 7.5, 45.3% females) underwent TMS-EEG evaluation of L-DLPFC and R-DLPFC with Delphi-MD and predictability of cognitive performance by demographics and clinical features.

### Associations of demographic and clinical features with cognitive scores

**Figure 2:**
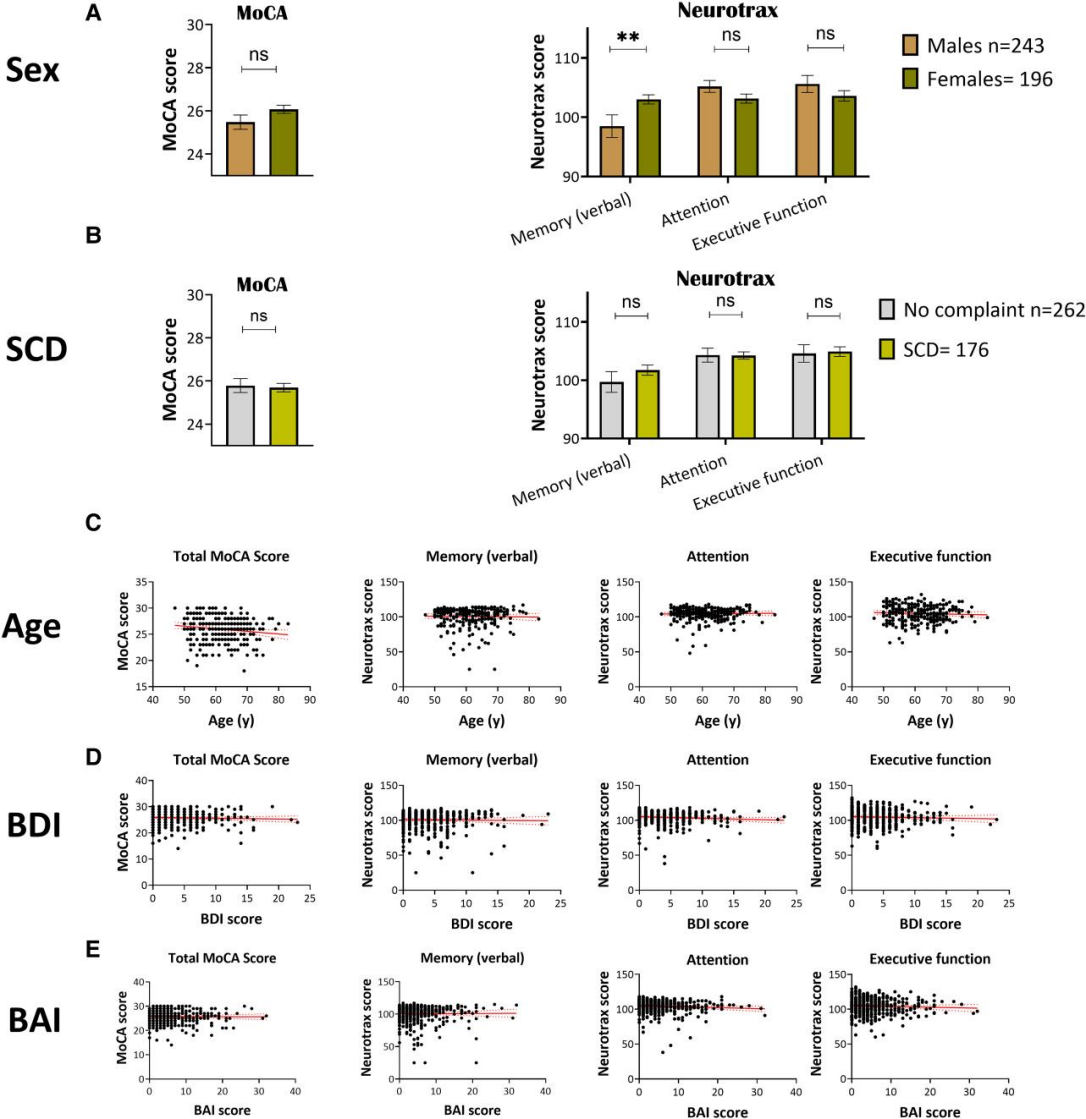
**Relation of demographic and clinical features to cognitive performance. (A**) bar graphs of the mean ±95% CI of MoCA, computerized memory, attention and executive function score in males versus females (with only memory significant following Bonferroni correction of alpha level to account for multiple comparisons (*P* < 0.012): MoCA: *t* = −2.3, *P* = 0.019; memory: *t* = −3.5, *P* < 0.001; attention: *t* = 2.3, *P* = 0.021; executive function: *t* = 1.7, *P* = 0.075). (**B**) bar graphs of the mean ±95% confidence interval of MoCA, computerized memory, attention and executive function score in subjects with no subjective cognitive complaints (grey) versus subjective cognitive decline (light green) (MoCA: t = 0.36, *P* = 0.719; memory: *t* = −1.5, *P* = 0.12; attention: *t* = 0.07, *P* = 0.944; executive function: *t* = −0.28, *P* = 0.773). Levels of significance: * marks *P* < 0.05, ** marks < 0.01, ns = not significant. (**C**) scatter plots, of individual subjects, describing the linear regression line ±95% confidence interval of subjects’ age compared to cognitive performance (MoCA (*F*(1,436) = 23.4, *P* < 0.001, *R*^2^ = 0.051), memory (*F*(1,437) = 1.11, *P* = 0.292, *R*^2^ = 0.003), attention (*F*(1,437) = 0.003, *P* = 0.955, *R*^2^ = 0.000), executive function (*F*(1,437) = 0.93, *P* = 0.334, *R*^2^ = 0.002]). (**D**) scatter plots, of individual subjects, describing the linear regression line ±95% confidence interval of subjects’ BDI (Beck’s Depression Inventory) score compared to cognitive performance (MoCA [*F*(1,425) = 0.72, *P* = 0.396, *R*^2^ = 0.002], memory (*F*(1,426) = 0.159, *P* = 0.690, *R*^2^ = 0.000), attention (*F*(1,426) = 3.99, *P* = 0.046, *R*^2^ = 0.009), executive function (*F*(1,426) = 0.91, *P* = 0.341, *R*^2^ = 0.002)). (**E**) scatter plots, of individual subjects, describing the linear regression line ±95% confidence interval of subjects’ BAI (Beck’s Anxiety Inventory) score compared to cognitive performance (MoCA (*F*(1,428) = 0.03, *P* = 0.869, *R*^2^ = 0.000), memory (*F*(1,429) = 0.08, *P* = 0.772, *R*^2^ = 0.000), attention (*F*(1,429) = 3.39, *P* = 0.06, *R*^2^ = 0.008) and executive function (*F*(1,429) = 1.46, *P* = 0.227, *R*^2^ = 0.003)).

A multivariate linear regression model was applied to detect possible demographic or clinical predictors of cognitive performance. To that end, age, sex, depression score (BDI), anxiety score (BAI) and subjective report of cognitive complaints were combined as possible predictors of cognitive performance scores, as they are considered to be known risks for cognitive impairments. The linear regression model successfully predicted MoCA score (F (5421) = 5.71, *P* < 0.001, adjusted R^2^ = 0.053). This was driven predominantly by age, which was the only significant factor (*P* < 0.001). A successful prediction of memory (computerized verbal, Neurotrax) was also observed (*F*(5,422) = 2.67, *P* = 0.021, adjusted R^2^ = 0.019), this was driven by sex (*P* < 0.001), no other factors significantly predicted cognitive performance scores. None of the tested factors (age, sex, BDI, BAI and subjective report of cognitive complaints) were able to successfully predict attention or executive function scores (computerized tests, Neurotrax) (*F*(5,422) = 2.05, *P* = 0.071, adjusted R^2^ = 0.012; *F*(5,422) = 1.44, *P* = 0.210, adjusted *R*^2^ = 0.005, respectively). Figure 2 presents each clinical, demographic category (sex, cognitive complaint) and continuous measure (age, BDI score, BAI score) separately, versus each of the cognitive scores (MoCA total score, memory (verbal), attention and executive function). The full regression models results can be found in Supplementary Tables 1–4.

### Clustering by cognitive performance:

**Figure 3:**
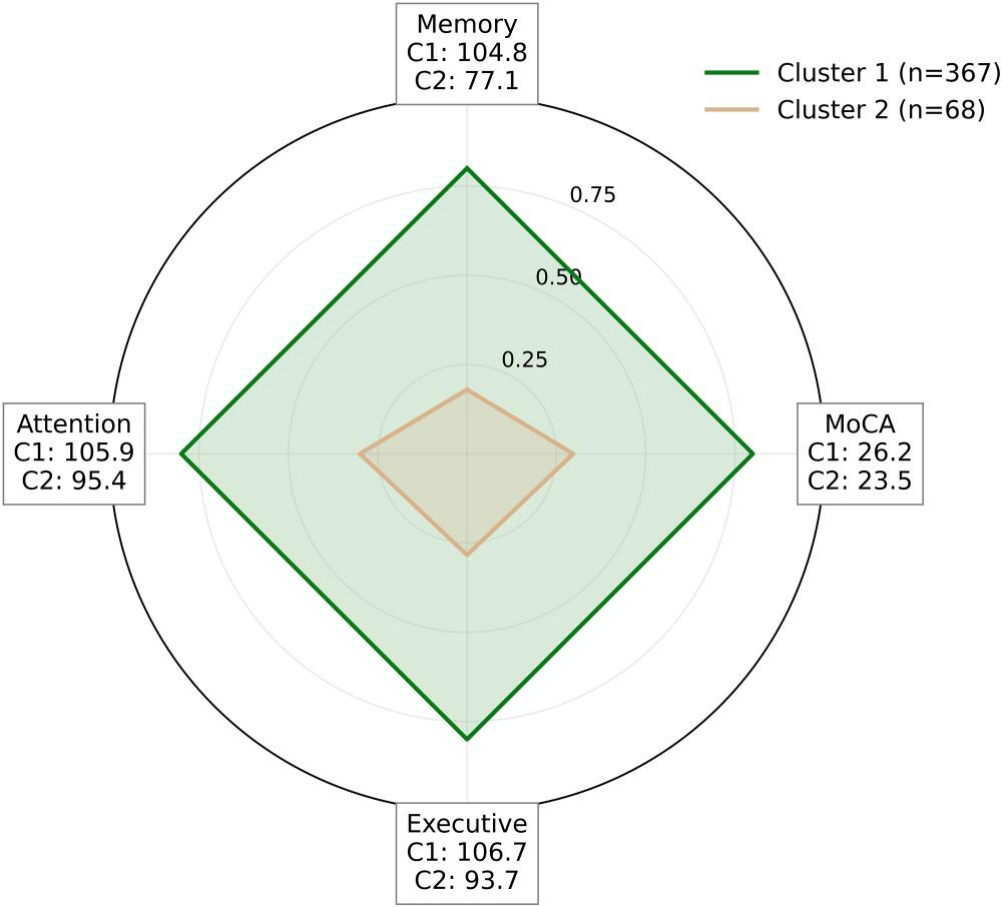
**Cognitive scores centroids of each *k*-means cognitive clusters.** Cluster 1 (green) entails higher scores of total MoCA score, computerized (Neurotrax) executive function, attention and memory (verbal) compared to Cluster 2 (tan) (ANOVA, *F*(1,434) = 64.7, *F*(1,434) = 75.3, *F*(1,434) = 74.0, *F*(1,434) = 580.8, respectively, all *P* < 0.001). (ANOVA, all *P* < 0.001). The *F* tests should be used only for descriptive purposes because the clusters have been chosen to maximize the differences among cases in different clusters.

To test for cognitive performance clustering and create a unified, single comprehensive cognitive profile, a *k*-means cluster analysis was applied, including total MoCA score and computerized testing scores of verbal memory, attention and executive function. With *k* = 2, the algorithm converged after iteration 9 (maximum absolute centre change = 0.000; Minimum distance between initial centres = 121.848). Of 454 records, 435 were analysed (19 listwise-missing either one of the cognitive scores), yielding two cognitive clusters. Cluster 1: CN, *n* = 367 and Cluster 2: CI, *n* = 68. Final centroids (means) indicated a consistently higher cognitive profile in Cluster 1 versus Cluster 2: MOCA 26.17 versus 23.45, Memory (verbal) 104.79 versus 76.69, Attention 105.77 versus 95.81, and Executive function 106.68 versus 93.72 (Fig. 3). The clusters differed in MoCA score and all three cognitive domains (ANOVA, all *P* < 0.001). The *F* tests should be used only for descriptive purposes because the clusters have been chosen to maximize the differences among cases in different clusters. The clusters did not differ in demographic and clinical features including age, education, BDI score, BAI score or subjective cognitive complaint (*P* = 0.331, *P* = 0.596, *P* = 0.872, *P* = 0.987, *P* = 0.107, respectively) but did display lower proportion of females in the CI cluster (30.6%) compared to the CN cluster (47.9%) (*P* = 0.007) (Table 1). In terms of the clusters’ RMT no differences were observed for either left- or right-hand RMT (*P* = 0.62 and *P* = 0.59, respectively). CI cluster measured with a mean RMT of 77.0% ± 12.4 STD and 73.7% ± 12.5 STD of device maximal capacity for right and left hands, respectively, while for CN cluster a mean 76.0% ± 11.1 STD and 75.0% ± 12.3 STD for right and left hands, respectively.

**Table 1:** Cognitive clusters’ characteristics

	Cluster				
	CI	CN	t	df	*P*-value
*N*	68	367			
Sex (% Females)	30.6%	47.9%			0.007
Age (Mean ± SD)	62.19 ± 6.9	61.3 ± 7.6	0.976	106.352	0.331
Years of education (Mean ± SD)	16.2 ± 2.6	16.5 ± 3.0	−0.534	52.298	0.596
BDI score (Mean ± SD)	4.1 ± 4.3	4.0 ± 3.7	0.161	89.574	0.872
BAI score (Mean ± SD)	5.4 ± 5.9	5.4 ± 5.4	−0.017	93.163	0.987
Total MOCA Score (Mean ± SD)	23.5 ± 3.0	26.1 ± 2.3	−6.772	88.473	<0.001
Memory (verbal) (Mean ± SD)	77.4 ± 16.6	105.0 ± 6.2	−13.856	75.077	<0.001
Attention (Mean ± SD)	96.3 ± 14.6	105.8 ± 6.7	−5.396	77.101	<0.001
Executive function (Mean ± SD)	94.5 ± 12.8	106.6 ± 10.4	−7.527	90.255	<0.001
Subjective cognitive complaint (% of total)	31.90%	42.10%			0.107

### Interhemispheric connectivity (TMS-EEG) and cognitive performance

**Figure 4:**
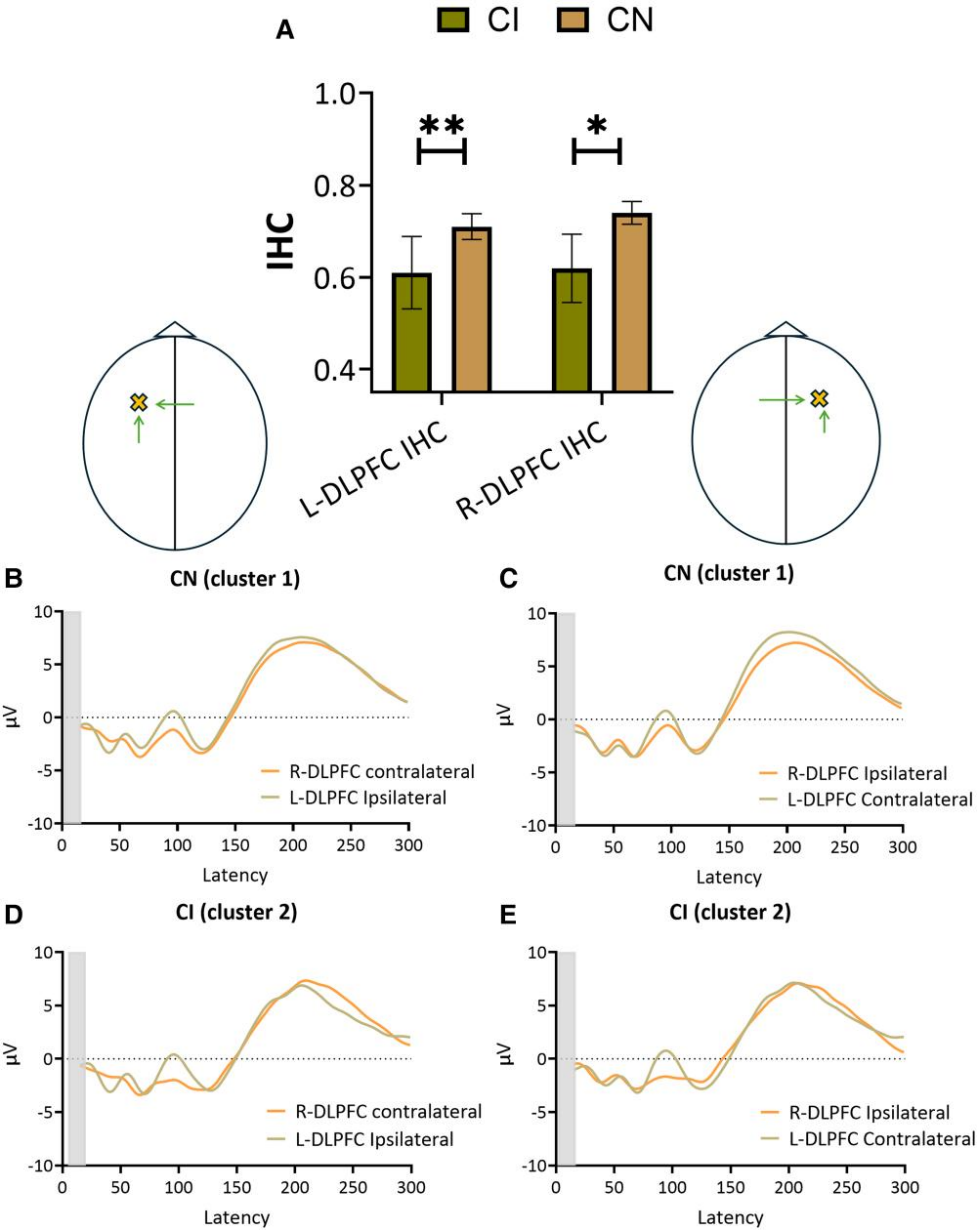
**IHC in CI and CN clusters and their TEP waveforms.** (**A**) Mean ±95% CI of L-DLPFC and R-DLPFC IHC of cognitive clusters: CN (*n* = 367, brown), CI (*n* = 68, blue) (Mann–Whitney U-test: R-DLPFC: U = 16 306, *P* < 0.001; L-DLPFC: U = 15 692, *P* = 0.001). (**B**) Presentation of the mean TEP waveform of L-DLPFC left hemisphere electrodes in response to left stimulation (light Khaki) versus the right stimulation (orange) within the CN cluster. (**C**) Presentation of the Mean TEP waveform of R-DLPFC right hemisphere electrodes in response to left stimulation (dark Khaki) versus the right stimulation (green) within the CN cluster. (**D**) Presentation of the Mean TEP waveform of L-DLPFC left hemisphere electrode in response to left stimulation (light Khaki) versus the right stimulation (orange) within the CI cluster. (**E**) Presentation of the Mean TEP waveform of R-DLPFC right hemisphere electrode in response to left stimulation (dark Khaki) versus the right stimulation (green) within the CI cluster. *P < 0.05, **P < 0.01.

IHS interaction with cognitive performance was tested by comparing IHC index between the two clusters. The Mann–Whitney tests indicated that IHC was significantly lower in the CI cluster (*n* = 68) compared with the CN cluster (*n* = 367). For the R-DLPFC, CI participants had a mean IHC of 0.63 ± 0.32 versus 0.75 ± 0.25 in CN group (U = 16,306, *P* < 0.001). For the L-DLPFC, CI participants showed a mean IHC of 0.61 ± 0.34 compared with 0.71 ± 0.28 in CN (U = 15,692, *P* = 0.001). These findings confirm a robust group-level decrease in DLPFC IHC associated with cognitive impairment (Fig. 4).

As the results demonstrated highest association values for IHC, age and sex with cognitive status, we sought out to explore their combined predictability of cognitive performance clusters (CI versus CN) using binary logistic regression. The overall model including R-DLPFC was significant (χ^2^ (3) = 18.85, *P* < 0.001), explaining 4.3–7.3% of the variance (Cox & Snell *R*^2^ = 0.043; Nagelkerke *R*^2^ = 0.073).

**Figure 5:**
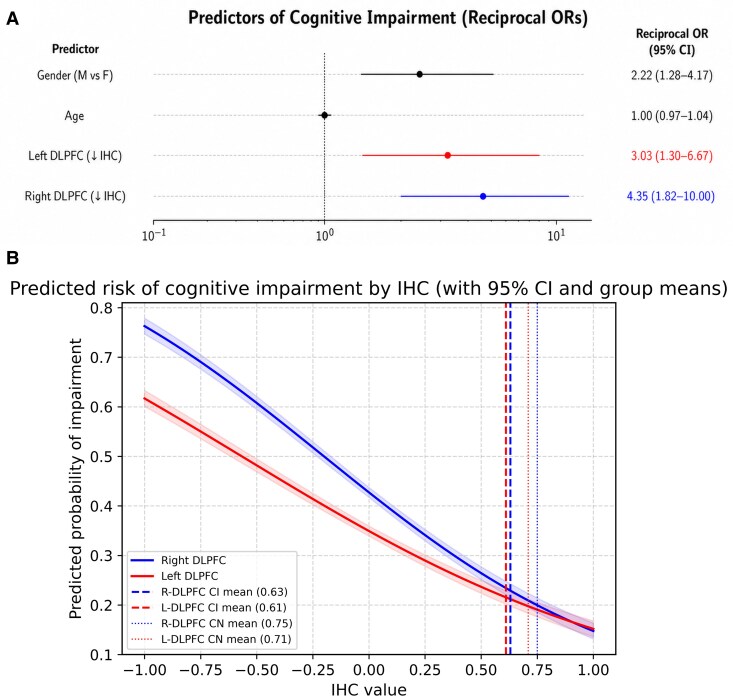
**Associations between DLPFC IHC and cognitive impairment.** (**A**) Forest plots of odds ratios (OR) from binary logistic regression models for age, sex (female versus male) with L-DLPFC IHC (red) or R-DLPFC IHC (blue) (R-DLPFC model: χ^2^(3) = 18.85, *P* < 0.001; L-DLPFC model: χ^2^(3) = 14.84, *P* = 0.002; L-DLPFC IHC: *P* = 0.008; R-DLPFC IHC: *P* < 0.001; sex: *P* = 0.006; age: *P* > 0.89) in predicting cognitive cluster (*n*(CN) = 367, *n*(CI) = 68). Left and right IHC and sex were significant predictors of cognitive impairment, whereas age showed no effect. Reciprocal OR values >1 indicate increased risk of impairment. (**B**) Predicted probability curves, based on the previous logistic regression model, of cognitive impairment by IHC values for L-DLPFC (red) and R-DLPFC (blue), with 95% confidence intervals (shaded areas). Vertical dashed lines mark the mean IHC values of cognitively impaired (CI, *n* = 68) and cognitively normal (CN, *n* = 367) groups. Lower IHC values were associated with higher predicted risk of impairment, consistent across both hemispheres (L-DLPFC: *P* = 0.008; R-DLPFC: *P* < 0.001).

Lower R-DLPFC IHC was associated with substantially increased odds of cognitive impairment (B = −1.46, SE = 0.43, *P* < 0.001). Specifically, a one-unit decrease in R-DLPFC IHC corresponded to approximately 4.35-fold higher odds of impairment (reciprocal OR = 4.35, 95% CIboot = 1.82–10.00). The overall model with L-DLPFC was significant (χ^2^(3) = 14.84, *P* = 0.002), explaining 3.4–5.8% of the variance (Cox & Snell R^2^ = 0.034; Nagelkerke R^2^ = 0.058). Lower L-DLPFC IHC was associated with increased odds of cognitive impairment (B = −1.10, SE = 0.41, *P* = 0.008), with a one-unit decrease corresponding to 3.03-fold higher odds (reciprocal OR = 3.03, 95% CIboot = 1.30–6.67). For both R-DLPFC and L-DLPFC IHC models, sex was able to predict impairment, with males having ∼2.2× higher odds of impairment versus females (Reciprocal OR = 2.22, 95% CIboot = 1.28–4.17, *P* = 0.006). Interestingly in both the L- DLPFC and R- DLPFC models, age was not a significant predictor (L-DLPFC: OR = 1.00, 95% CIboot ≈ 0.97–1.04, *P* = 0.89; R-DLPFC: OR = 1.00, 95% CIboot ≈ 0.97–1.04, *P* = 0.93), suggesting that once IHS is accounted for, chronological age does not independently differentiate cognitive cluster membership (Fig. 5A). A sensitivity analysis did not reveal a major change to results, when including years of education as a factor in the logistic regression model. Full results are presented in Supplementary Table 5.

In addition, binary logistic regression showed that lower IHC values in both L-DLPFC and R-DLPFC were associated with higher predicted probability of cognitive impairment, with group means for the impaired cluster falling at significantly lower IHC values compared to the CN cluster (Fig. 5B).The sample was stratified by sex to verify that the predictive value of DLPFC and IHC did not differ between males and females. In males, both L-DLPFC and R-DLPFC IHC significantly predicted cognitive impairment, with higher IHC associated with reduced odds of impairment (Left OR = 5.13, *P* = 0.006; Right OR = 3.99, *P* = 0.008), while age was not significant. In females, the effect was somewhat weaker but remained significant for both hemispheres (Left OR = 3.54, *P* = 0.049; Right OR = 3.54, *P* = 0.049), again independent of age. Importantly, the IHC × sex interaction term was not significant for either hemisphere (*P* > 0.6), confirming that the predictive effect of DLPFC IHC was consistent across sex and not attributable to the higher prevalence of impairment among males in this cohort.

## Discussion

In this large community-dwelling cohort, we demonstrate that reduced IHC within the DLPFC is related to higher odds of cognitive impairment. Logistic regression models showed that lower IHC values in both the L-DLPFC and R-DLPFC predicted higher odds of impairment, with the probability curves approaching 70–75% risk at the lowest IHC levels. In contrast, higher IHC values were linked to substantially reduced risk, emphasizing the potential of IHC as a robust marker of preserved cognitive function. These associations remained significant after adjustment for age and sex, supporting the specificity of IHC as a neurophysiological indicator of cognitive vulnerability. These results align with prior evidence linking interhemispheric integration to executive and memory performance^39^ and extend this work by quantifying the risk profile across a physiological range of IHC values. The effect was somewhat stronger for the right hemisphere but presented bilaterally, reinforcing the role of disrupted prefrontal connectivity as a neurophysiological marker of impaired cognition. Examining the left and right inter-intra hemispheric connectivity, separately, was of interest, as there is evidence that a rightward shift in prefrontal networks occurs in MCI and Ad patients.^40^ We sought to explore whether such lateralized patterns could be detected in a community-dwelling population using TMS-EEG, and whether a specific direction of IHS would be preferentially associated with cognitive impairment. In the present study, associations were observed for both hemispheres, suggesting that IHS in either direction may be relevant for cognitive function rather than reflecting a strictly lateralized effect. Sex differences, while significant, did not drive the effect of IHC. The precision of estimates was enhanced using bootstrapping across this large cohort, strengthening confidence in the robustness of the effect. Therefore, it is reasonable to assume that in a neurological care setting or a memory clinic where the prevalence and severity of the impairments is higher, IHC may exhibit higher performance. TMS-EEG provides a causal, time-resolved probe of cortical effective connectivity by perturbing one node and measuring the distributed evoked response, allowing interrogation of circuit communication rather than mere correlation.^3,41,42^ The DLPFC is a strategic hub for executive control, working memory and top-down modulation; Reduced interhemispheric coordination across prefrontal cortices is a well-described feature of ageing and cognitive impairment, often interpreted within compensation/de-differentiation frameworks such as HAROLD (Hemispheric Asymmetry Reduction in Older Adults).^43^ As MoCA captures global cognitive status,^3^ the convergence of lower IHC with both MoCA and domain-specific deficits strengthens the case that IHC tags clinically meaningful impairment rather than isolated test variance. In this study, we related to cognitive impairment by defining relative cognitive performance profiles derived from data-driven clustering rather than clinically defined diagnoses. This approach was chosen to capture cognitive heterogeneity within a healthy population, where performance is expected to vary along a continuum. Accordingly, findings should be interpreted at the population level, with limited direct clinical diagnostic applicability. In our results, the R-DLPFC demonstrated a steeper decrease in the cognitively impaired individuals compared to the left one. Notably, a study that tested the effects of rTMS on L-DLPFC and R-DLPFC found that it was the R-DLPFC inhibition that enhanced recognition memory in both HCs and MCIs, while excitatory R-DLPFC rTMS-caused deteriorated memory performance in HC.^44^ This was reinforced in a later study performed in Alzheimer’s disease patients, where it was the R-DLPFC inhibitory stimulation that contributed to increase in cognitive performance compared to sham.^45^

An imaging based study that examined structural and functional MRI in MCI patients found that bilateral DLPFC showed reduced functional connectivity with other prefrontal and related subcortical regions (which also correlated to cognitive performance) in addition to enhanced functional connectivity between the L-DLPFC and R-DLPFC.^46^ This pattern is consistent with the reduced IHC observed here, reflecting a larger discrepancy between intra- and inter-DLPFC connectivity in the cognitively impaired cluster. A longitudinal study found that prefrontal fMRI activation increases were associated with longitudinal declines in WM microstructure in a portion of the corpus callosum connecting the increasingly recruited frontal regions.^47^ These studies provide additional support and evidence to further solidify our observation that effective connectivity in the DLPFC represented here by lower DLPFC IHC are predictive of poorer cognitive profiles (lower MoCA and domain scores).

In the current study, recognized clinical and demographic risk correlates such as depressive and anxiety symptoms, and subjective cognitive complaint explained little variance in MoCA and computerized cognitive scores, with age driving the prediction of MoCA score and sex driving prediction of memory scores. The non-significant effect of age in prediction of computerized cognitive scores could be explained by the fact that these are normalized scores adjusted to age and education. In our sample, males generally demonstrated lower memory scores. This is consistent with published results of a large study of 26 088 participants with 8 years of follow-up, where women, compared with men, had higher baseline performance in global cognition, executive function, and memory. Although women had faster declines in global cognition and executive function, this was not the case for memory.^48^ Although, significant differences were found between males and females’ prevalence in our constructed cognitive clusters, controlling for sex and age did not reverse the decrease in IHC in either R-DLPFC or L-DLPFC, nor did sex have any effect when defined as the predictor. Thus, IHC differences between CN and CI subjects were consistent across sexes.

Despite their modest magnitude, the nearly identical left–right DLPFC IHC correlations (Supplementary Fig. 1) strengthen the bilateral reliability of the measure, while their relatively weak association with behavioural cognitive tests underscores that IHC captures a distinct physiological dimension of cortical efficiency beyond task performance. Clinically, an objective, rapid, non-invasive biomarker that indexes cortical network integrity is imperative for risk stratification, triage, and tracking change. IHC could complement cognitive screening and fluid biomarkers by improving detection sensitivity in individuals with borderline scores or variable results, and may help adjudicate mixed clinical representations (e.g. mood symptoms co-occurring with subtle cognitive change). As the clinical practice moves towards standard usage of fluid biomarker in symptomatic cases, utilizing IHC can potentially shed direct information on brain physiological function that are effected by the pathology, and provide an objective measure of serial monitoring those changes or dynamic effects of treatments targeting those pathological agents.

The persistence of the IHC effect after age/sex adjustment suggests that IHC is not merely a proxy for ageing or sex differences but may index circuit-level efficiency that may help predict MCI in advance of its onset. Thus, because TMS-EEG can be repeated over time and is sensitive to plasticity, IHC may be useful for monitoring interventions designed to enhance prefrontal network function (e.g. cognitive training, neuromodulation, or risk-factor modification). Limitations of the study include the cross-sectional design (precluding causal inference or prognostication). This precludes causal inference to the determination of whether reduced IHC precedes evident cognitive decline and may serve as an early marker. Long-term follow-ups are on their way and will be reported in future publications. Future longitudinal work would test whether lower DLPFC IHC predicts incident cognitive decline and whether interventions that increase IHC can improve performance, key criteria for qualification as a surrogate indicator of risk for cognitive deterioration or dementia and determine clear-cut thresholds. Adding multimodal comparators (structural/functional imaging, blood biomarkers) as well as everyday functional outcomes would also clarify the unique and shared variance captured by TMS-EEG IHC. While other health related confounders were collected (e.g. vascular risk, metabolic status, sleep quality) and examined exploratorily, they were not incorporated in the primary analyses to avoid overfitting and multicollinearity in the context of the current sample size. Future studies with larger cohorts and longitudinal designs should explicitly integrate these domains to refine the specificity of IHC–cognition associations. Additionally, in a future study we aim to measure the test retest of the IHC to further substantiate the bounds of a meaningful change. Finally, an external validation with an independent sample will be required to corroborate these results.

In summary, reduced DLPFC IHS measured with TMS-EEG represents a reproducible neurophysiological signature of cognitive vulnerability, aligning with contemporary models of network-level ageing and impairment, and supporting IHC as a promising objective indicator for dementia risk stratification, monitoring cognitive decline and effect of intervention.

## Supplementary Material

fcag251_Supplementary_Data

## Data Availability

Data supporting the findings of this study are available from the corresponding author upon reasonable request.
