# Supplementary material for: Interhemispheric connectivity measured with transcranial magnetic stimulation and EEG as an objective marker of cognitive risk
Source: Brain Commun. 2026 Jul 15;8(4):fcag251. doi: 10.1093/braincomms/fcag251 (PMC13372024; doi:10.1093/braincomms/fcag251)
Supplement: fcag251_Supplementary_Data [file fcag251_supplementary_data.docx]

**Supplementary materials:**

**Supplementary Table 1- Linear Regression of age, sex, depression scores, anxiety scores and subjective cognitive decline (SCD) versus MoCA total score:**

| **Model Summary** | | | | |
| --- | --- | --- | --- | --- |
| Model | R | R Square | Adjusted R Square | Std. Error of the Estimate |
| 1 | .253^a^ | 0.064 | 0.053 | 2.59375 |
| a. Predictors: (Constant), BAI, Age, SCD, Sex, BDI | | | | |

| **ANOVA^a^** | | | | | | |
| --- | --- | --- | --- | --- | --- | --- |
| Model | | Sum of Squares | df | Mean Square | F | p-value |
| 1 | Regression | 192.183 | 5 | 38.437 | 5.713 | <0.001^b^ |
|  | Residual | 2798.663 | 416 | 6.728 |  |  |
|  | Total | 2990.846 | 421 |  |  |  |
| a. Dependent Variable: Total MoCA score | | | | | | |
| b. Predictors: (Constant), BAI, Age, SCD, Sex, BDI | | | | | | |

| **Coefficients^a^** | | | | | | |
| --- | --- | --- | --- | --- | --- | --- |
| Model | | Unstandardized Coefficients | | Standardized Coefficients | t | p-value |
|  |  | B | Std. Error | Beta |  |  |
| 1 | (Constant) | 29.973 | 1.162 |  | 25.799 | <0.001 |
|  | Age | -0.078 | 0.017 | -.221 | -4.594 | <0.001 |
|  | Sex | 0.515 | 0.268 | .096 | 1.920 | 0.055 |
|  | SCD | -0.048 | 0.269 | -0.009 | -0.180 | 0.857 |
|  | BDI | -0.030 | 0.041 | -0.043 | -0.733 | 0.464 |
|  | BAI | -0.008 | 0.028 | -0.017 | -0.292 | 0.771 |
| a. Dependent Variable: Total MoCA score | | | | | | |

**Supplementary Table 2- Linear Regression of age, sex, depression scores, anxiety scores and subjective cognitive decline (SCD) versus Neurotrax^TM^ Memory (verbal) score:**

| **Model Summary** | | | | |
| --- | --- | --- | --- | --- |
| Model | R | R Square | Adjusted R Square | Std. Error of the Estimate |
| 1 | 0.176^a^ | 0.031 | 0.019 | 12.96752 |
| a. Predictors: (Constant), BAI, Age, SCD, gender, BDI | | | | |

| **ANOVA^a^** | | | | | | |
| --- | --- | --- | --- | --- | --- | --- |
| Model | | Sum of Squares | df | Mean Square | F | p-value |
| 1 | Regression | 2251.004 | 5 | 450.201 | 2.677 | 0.021^b^ |
|  | Residual | 70121.270 | 417 | 168.157 |  |  |
|  | Total | 72372.274 | 422 |  |  |  |
| a. Dependent Variable: Memory (verbal) | | | | | | |
| b. Predictors: (Constant), BAI, Age, SCD, gender, BDI | | | | | | |

| **Coefficients^a^** | | | | | | |
| --- | --- | --- | --- | --- | --- | --- |
| Model | | Unstandardized Coefficients | | Standardized Coefficients | t | p-value |
|  |  | B | Std. Error | Beta |  |  |
| 1 | (Constant) | 98.291 | 5.798 |  | 16.953 | <0.001 |
|  | Age | -0.052 | 0.085 | -.030 | -.610 | 0.542 |
|  | Sex | 4.267 | 1.335 | 0.162 | 3.197 | 0.001 |
|  | SCD | 1.184 | 1.346 | 0.044 | 0.880 | 0.379 |
|  | BDI | -0.240 | 0.204 | -0.070 | -1.176 | 0.240 |
|  | BAI | -0.019 | 0.142 | -0.008 | -0.132 | 0.895 |
| a. Dependent Variable: Memory (verbal) | | | | | | |

**Supplementary Table 3- Linear Regression of age, sex, depression scores, anxiety scores and subjective cognitive decline (SCD) versus Neurotrax^TM^ Attention score:**

| **Model Summary** | | | | |
| --- | --- | --- | --- | --- |
| Model | R | R Square | Adjusted R Square | Std. Error of the Estimate |
| 1 | 0.155^a^ | 0.024 | 0.012 | 8.88404 |
| a. Predictors: (Constant), BAI, Age, SCD, sex, BDI | | | | |

| **ANOVA^a^** | | | | | | |
| --- | --- | --- | --- | --- | --- | --- |
| Model | | Sum of Squares | df | Mean Square | F | p-value |
| 1 | Regression | 808.911 | 5 | 161.782 | 2.050 | 0.071^b^ |
|  | Residual | 32912.186 | 417 | 78.926 |  |  |
|  | Total | 33721.097 | 422 |  |  |  |
| a. Dependent Variable: Attention | | | | | | |
| b. Predictors: (Constant), BAI, Age, SCD, Sex, BDI | | | | | | |

| **Coefficients^a^** | | | | | | |
| --- | --- | --- | --- | --- | --- | --- |
| Model | | Unstandardized Coefficients | | Standardized Coefficients | t | p-value |
|  |  | B | Std. Error | Beta |  |  |
| 1 | (Constant) | 110.833 | 3.972 |  | 27.902 | <0.001 |
|  | Age | -0.047 | 0.059 | -0.040 | -0.811 | 0.418 |
|  | Sex | -1.984 | 0.914 | -0.111 | -2.170 | 0.031 |
|  | SCD | 0.546 | 0.922 | 0.030 | 0.592 | 0.554 |
|  | BDI | -0.176 | 0.140 | -0.075 | -1.254 | 0.210 |
|  | BAI | -0.037 | 0.097 | -0.023 | -0.381 | 0.703 |
| a. Dependent Variable: Attention | | | | | | |

**Supplementary Table 4- Linear Regression of age, sex, depression scores, anxiety scores and subjective cognitive decline (SCD) versus Neurotrax^TM^ Executive Function score:**

| **Model Summary** | | | | |
| --- | --- | --- | --- | --- |
| Model | R | R Square | Adjusted R Square | Std. Error of the Estimate |
| 1 | 0.130^a^ | 0.017 | 0.005 | 11.65753 |
| a. Predictors: (Constant), BAI, Age, SCD, Sex, BDI | | | | |

| **ANOVA^a^** | | | | | | |
| --- | --- | --- | --- | --- | --- | --- |
| Model | | Sum of Squares | df | Mean Square | F | p-value |
| 1 | Regression | 976.938 | 5 | 195.388 | 1.438 | 0.210^b^ |
|  | Residual | 56669.450 | 417 | 135.898 |  |  |
|  | Total | 57646.388 | 422 |  |  |  |
| a. Dependent Variable: Executive function | | | | | | |
| b. Predictors: (Constant), BAI, Age, SCD, Sex, BDI | | | | | | |

| **Coefficients^a^** | | | | | | |
| --- | --- | --- | --- | --- | --- | --- |
| Model | | Unstandardized Coefficients | | Standardized Coefficients | t | p-value |
|  |  | B | Std. Error | Beta |  |  |
| 1 | (Constant) | 116.046 | 5.212 |  | 22.264 | <0.001 |
|  | Age | -0.129 | 0.077 | -0.082 | -1.678 | 0.094 |
|  | Sex | -2.197 | 1.200 | -0.094 | -1.831 | 0.068 |
|  | SCD | 0.791 | 1.210 | 0.033 | 0.654 | 0.514 |
|  | BDI | -0.040 | 0.184 | -0.013 | -0.215 | 0.830 |
|  | BAI | -0.075 | 0.127 | -0.036 | -0.585 | 0.559 |
| a. Dependent Variable: Executive function | | | | | | |

**Supplementary Figure 1- Correlation matrix of cognitive scores and Interhemispheric connectivity IHC for left and right dorsolateral prefrontal cortex** (DLPFC):


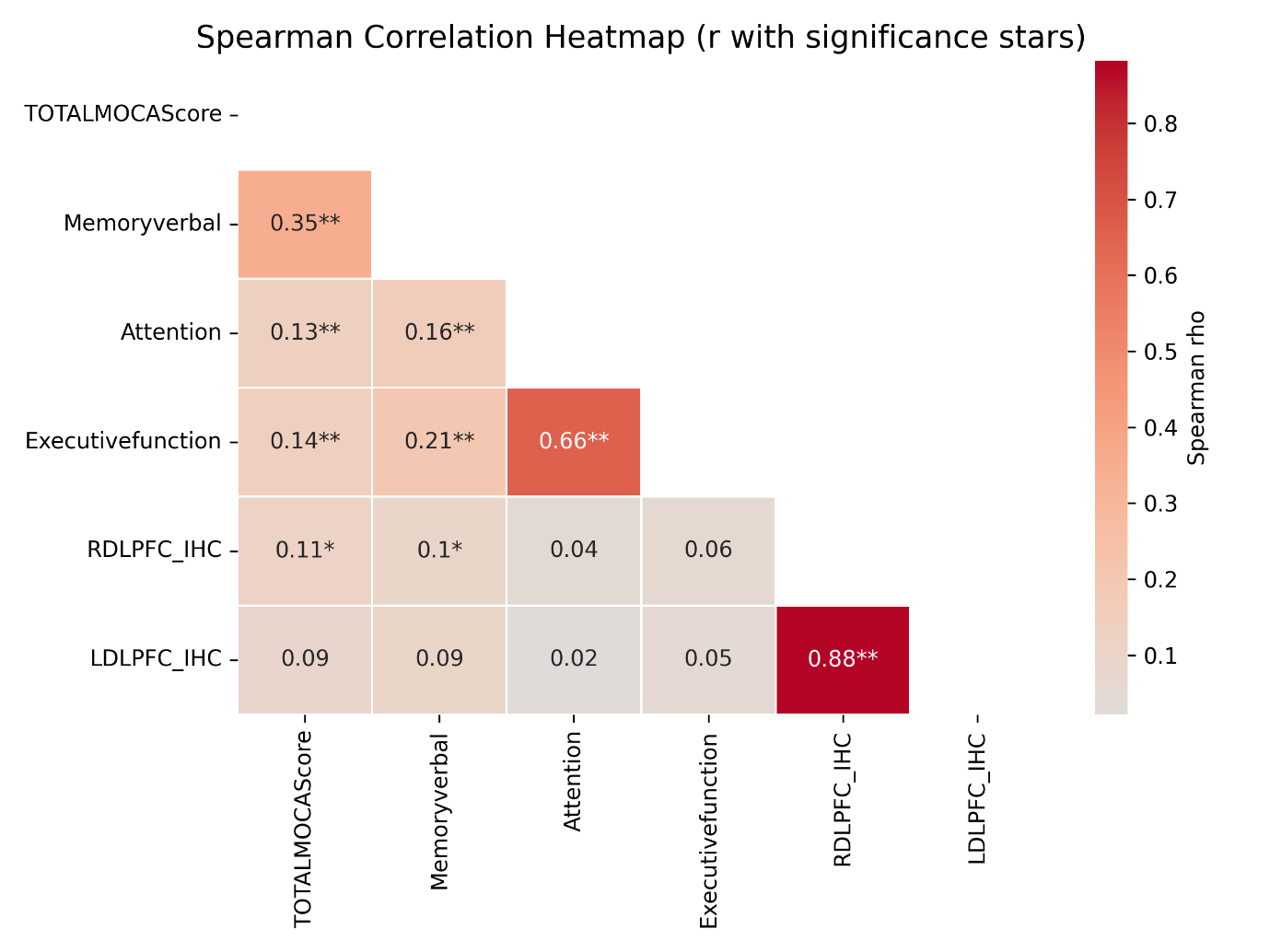


Figure 1- correlation matrix of left and right interhemispheric connectivity (IHC) and cognitive scores (MoCA, memory, attention and executive function) (N=435). Color codded Spearman r coefficients (higher r indicated by darker red color). *p-value<0.05, **p-value<0.01.

**Supplementary Table 5- Logistic Regression of age, sex, education and L-DLPFC or R-DLPFC IHC in prediction of cognitive cluster- A sensitivity analysis:**

1. **R-DLPFC IHC-**

| **Omnibus Tests of Model Coefficients** | | | | |
| --- | --- | --- | --- | --- |
|  | | Chi-square | df | p-value |
| Step 1 | Step | 19.100 | 4 | <0.001 |
|  | Block | 19.100 | 4 | <0.001 |
|  | Model | 19.100 | 4 | <0.001 |

| **Model Summary** | | | |
| --- | --- | --- | --- |
| Step | -2 Log likelihood | Cox & Snell R Square | Nagelkerke R Square |
| 1 | 363.326^a^ | 0.043 | 0.074 |
| a. Estimation terminated at iteration number 5 because parameter estimates changed by less than 0.001. | | | |

| **Variables in the Equation** | | | | | | | | | |
| --- | --- | --- | --- | --- | --- | --- | --- | --- | --- |
|  | | B | S.E. | Wald | df | p-value | Exp(B) | 95% CI for EXP(B) | |
|  |  |  |  |  |  |  |  | Lower | Upper |
| Step 1^a^ | Age | -0.002 | 0.018 | 0.009 | 1 | 0.926 | 0.998 | 0.964 | 1.034 |
|  | Sex (1) | -0.785 | 0.290 | 7.318 | 1 | 0.007 | 0.456 | 0.258 | 0.805 |
|  | Education years | -0.033 | 0.064 | 0.262 | 1 | 0.609 | 0.968 | 0.853 | 1.098 |
|  | R-DLPFC_IHC | -1.469 | 0.435 | 11.408 | 1 | <0.001 | 0.230 | 0.098 | 0.540 |
|  | Constant | 0.328 | 1.567 | 0.044 | 1 | 0.834 | 1.388 |  |  |
| a. Variable(s) entered on step 1: Age, Sex, Education years, R-DLPFC IHC. | | | | | | | | | |

1. **L-DLPFC IHC:**

| **Omnibus Tests of Model Coefficients** | | | | |
| --- | --- | --- | --- | --- |
|  | | Chi-square | df | p-value |
| Step 1 | Step | 15.063 | 4 | 0.005 |
|  | Block | 15.063 | 4 | 0.005 |
|  | Model | 15.063 | 4 | 0.005 |

| **Model Summary** | | | |
| --- | --- | --- | --- |
| Step | -2 Log likelihood | Cox & Snell R Square | Nagelkerke R Square |
| 1 | 367.363^a^ | 0.034 | 0.058 |
| a. Estimation terminated at iteration number 5 because parameter estimates changed by less than 0.001. | | | |

| **Variables in the Equation** | | | | | | | | | |
| --- | --- | --- | --- | --- | --- | --- | --- | --- | --- |
|  | | B | S.E. | Wald | df | p-value | Exp(B) | 95% CI for EXP(B) | |
|  |  |  |  |  |  |  |  | Lower | Upper |
| Step 1^a^ | Age | -0.001 | 0.018 | 0.004 | 1 | 0.952 | 0.999 | 0.964 | 1.035 |
|  | Sex (1) | -0.785 | 0.289 | 7.381 | 1 | 0.007 | 0.456 | 0.259 | 0.804 |
|  | Education years | -0.027 | 0.064 | 0.181 | 1 | 0.671 | 0.973 | 0.858 | 1.103 |
|  | L-DLPFC_IHC | -1.099 | 0.411 | 7.134 | 1 | 0.008 | 0.333 | 0.149 | 0.746 |
|  | Constant | -0.092 | 1.550 | 0.004 | 1 | 0.953 | 0.912 |  |  |
| a. Variable(s) entered on step 1: Age, Sex, Education years, L-DLPFC IHC. | | | | | | | | | |
